# Preparation of Antibacterial Gelatin/Genipin Nanofibrous Membrane for Tympanic Membrane Repair

**DOI:** 10.3390/molecules27092906

**Published:** 2022-05-03

**Authors:** Shuying Han, Zhaohua Zhang, Jia Chen, Jie Li, Mi Zhou, Zejian He, Zhen He, Longfei Li

**Affiliations:** 1 Material Science and Engineering, College of Materials Science and Engineering, Zhejiang University of Technology, Hangzhou 310000, China; 17826801650@163.com (S.H.); zhaohua119@126.com (Z.Z.); hezejian@zjut.edu.cn (Z.H.); hezhen0821@163.com (Z.H.); lilongfei9708@163.com (L.L.); 2 The Department of Otolaryngology, The Second Affiliated Hospital, School of Medicine, Zhejiang University, Hangzhou 310000, China; chenjia212c@zju.edu.cn; 3 Research and Development Department, Hangzhou Singclean Medical Products Co., Ltd., Hangzhou 310000, China; lijie@hzxhe.com

**Keywords:** gelatin, genipin, levofloxacin, nanofiber membrane, tympanic membrane

## Abstract

Tympanic membrane perforation (TMP), a common disease, often needs a scaffold as the patch to support surgery. Due to the environment of auditory meatus, the patch can be infected by bacteria that results in failure; therefore, the ideal scaffold may combine biomimetic and antibacterial features. In this work, gelatin was used as the electrospinning framework, genipin as the crosslinking agent, and levofloxacin as an antibacterial in order to prepare the scaffold for TMP. Different contents of levofloxacin have been added to gelatin/genipin. It was found that, with the addition of levofloxacin, the gelatin/genipin membranes exhibit improved hydrophilia and enhanced tensile strength. The antibacterial and cell-cultured experiments showed that the prepared antibacterial membranes had excellent antibacterial properties and good biocompatibility, respectively. In summary, levofloxacin is a good group for the gelatin/genipin scaffold because it improves the physical properties and antibacterial action. Compared with different amounts of levofloxacin, a gelatin/genipin membrane with 1% levofloxacin is more suitable for a TM.

## 1. Introduction

Tympanic membrane (TM) is a thin semi-transparent structure with three layers, including a keratinizing squamous epithelial outer layer, fibrous middle layer, and mucosal inner layer [1]. Due to otitis media or trauma, TM is easily perforated, which has long been regarded as the most common complication for oncological diseases [2].Without proper treatment, TM perforations may lead to increased susceptibility to infection and otorrhea, conductive hearing loss (CHL), associated speech problems, and cholesteatoma formation [3,4].

In order to treat TM perforations, a scaffold, including autologous tissue (fat, fascia, or perichondrium) [5,6,7] or a man-made patch (rice paper or Gelfoam) [8,9], is required in myringoplasty or tympanoplasty surgery. Autologous grafts have been normally utilized clinically with good success rates, while it requires invasive technique and great operative time [10,11,12]. With the development of tissue engineering, researchers have focused on man-made absorbable scaffolds [13,14,15,16]. Ideally, the scaffold material should provide a structure and an environment for cells to attach, expand and perform their normal functions [17]. Moreover, suitable mechanical properties and an antibacterial effect are also important for the scaffold because it must sustain the vibration of sound and faces the auditory meatus, an environment with bacteria [18,19,20].

It is well known that electrospinning has been widely used for the simple and continuous manufacture of nanofibers, and it has attracted more attention and interest in applications such as tissue engineering and drug delivery [21,22,23]. Electrospun scaffolds can be fabricated with nanoscale structures that are similar to the natural extracellular matrix [23]. Based on previous works, the electrospun gelatin nanofiber membrane that was crosslinked by genipin showed good mechanical properties, water-tolerance, and excellent biocompatibility [14,24,25,26,27]. However, the gelatin fiber membrane is susceptible to infection by bacteria and other microorganisms, which limits its application in tissue engineering. In order to enhance the antibacterial property, an antibacterial agent is often added to the gelatin film [28,29]. Levofloxacin is a prominent broad-spectrum antibacterial agent with low price and high efficacy [30]. The mechanism of action is mainly to inhibit the bacterial rotatable enzyme activity so that the replication and transcription of the bacteria are blocked, thus achieving the effect of killing bacteria [30,31].

In this work, electrospun gelatin/genipin/levofloxacin nanofibers were prepared as the scaffold for a TM repair. Scanning electron microscopy (SEM), attenuated total reflection Fourier transform infrared spectroscopy (ATR-FTIR), and water contact angle and tensile tests were adopted to characterize the morphology, chemical structure, wettability, and mechanical properties of the antibacterial gelatin/genipin/levofloxacin membrane. In addition, the proliferative response of umbilical vein endothelial cells and fibroblasts on the antibacterial gelatin nanofiber membrane was observed.

## 2. Materials and Methods

### 2.1. Materials

Gelatin (CAS: 9000-70-8, AR), genipin (CAS: 6902-77-8, ≥98%), acetic acid (CAS: 64-19-7, AR), levofloxacin (CAS: 100986-85-4, ≥98%), and other chemical reagents were purchased from Aladdin (Shanghai, China), and they were used as received without further purification.

### 2.2. Electrospinning Precursor

The electrospinning precursor for gelatin/genipin nanofibers was prepared in these typical steps. Firstly, gelatin (2 g) was added to the mixture solution of acetic acid (5 g) and deionized water (3 g) and stirred to obtain the gelatin solution. Subsequently, a certain amount of levofloxacin was added and stirred to be mixed. Afterwards, 0.08 g genipin was added into the gelatin/levofloxacin solution based on the mass proportion of gelatin (4 wt%) for acquiring the precursor. It should be noted that, in the experiment, because crosslinking and the addition of genipin started at the same time, the solution should be electrospun as soon as possible to prevent the influences of crosslinking on fiber morphology. After adding genipin, the mixed solution needs to be stirred for about 5 min to make genipin evenly mixed in the system.

### 2.3. Nanofibrous Membranes via Electrospinning

The precursor was poured into a 20 mL plastic syringe that was equipped with a copper needle with a diameter of 0.8 mm. The syringe was fixed on the micro-injection pump with an injection rate of 0.8 mL/h, and the needle was connected to high-voltage power (20 kV) supply electrospinning. A piece of flat aluminum foil was placed 10 cm from the tip of the needle to collect the nanofibers. The collected membrane was dried under a vacuum before being used at room temperature.

### 2.4. Crosslinking of Nanofibrous Membranes

The crosslinking reaction of genipin and gelatin required a high-humidity environment [32], while the gelatin could be dissolved in water, so the films should not be submerged in water. Therefore, the nanofibrous membranes were placed on aluminum foil in a vessel that was full of water vapor. It could be observed that the white gelatin fibrous membrane gradually turned blue. After 2 h, the membranes were dried under vacuum in a dryer with desiccant. The vacuum drying time is about 48 h in order to obtain a suitable membrane. Figure S1 (Supplementary Materials) provides the photograph of the membrane before (left) and after crosslinking (right).

### 2.5. Characterization

#### 2.5.1. Surface Tension Characterization

The surface tension of the precursor solution was checked by the pendant drop method on a video-based optical contact angle meter (Dataphysics OCA-20, Stuttgart, Germany). The solution drop volume of the test was controlled by a microsyringe, and the test conditions were kept at room temperature. It was measured at least 3 times and averaged on each sample surface. The conductivity of the solution was tested using a digital conductivity meter (Lei magnetosound Instrument, DDS-11A, Shanghai, China).

#### 2.5.2. Morphological Characterization

The whole sample preparation process was carried out at 25 °C and 50% relative humidity. After vacuum drying for 48 h, the fiber membranes were cut into 0.5 cm × 0.5 cm squares and coated with a thin layer of gold for SEM observation. The micro-morphology of each group’s membranes was photographed at 5000× and 10,000×. We have added a description of the experimental conditions in the relevant place of the revised manuscript. The morphology of the nanofibrous membranes was observed under scanning electron microscopy (SEM, S-4700, Hitachi Group Corp., Hitachi, Japan). Meanwhile, randomly selected areas were cut into squares and coated with a thin layer of gold.

#### 2.5.3. Fourier Transform Infrared Spectroscopy

The chemical structure of the nanofibrous membranes was characterized by the attenuated total reflectance Fourier transform infrared (ATR-FTIR) spectrophotometer (Thermo Fisher Scientific, Nicolet-6700, Waltham, MA, USA). Moreover, all spectra were taken directly in transmittance mode on a diamond ATR crystal cell in the spectral range of 4000~600 cm^−1^ by an accumulation of 64 scans with a resolution of 2 cm^−1^.

#### 2.5.4. Water Contact-Angle Measurement

The water contact angle value of the surface of nanofibrous membranes was measured with a video-based optical contact angle meter (Dataphysics OCA-20, Stuttgart, Germany) by the sessile drop method at room temperature. The samples were cut into pieces and placed on a glass slide with double-sided tape. The droplet was placed on the samples’ surface using a microgauge. Deionized water (2 µL) was automatically dropped on the surface of the samples to measure the angle degree. Each of the reported contact angles was obtained by averaging three values on different positions of the sample.

#### 2.5.5. Mechanical Properties Test

The INSTRON 5966 testing machine (INSTRON, Boston, MA, USA) was used to measure the applied load versus the nanofibrous membranes cut in 10 mm × 30 mm to calculate stress-strain curves. The thickness of the samples was measured by a digital caliper with a precision of 0.01 mm and then mounted in tensile grips. A 500 N load cell was used to test the samples at a rate of 20 mm/min. The test was conducted at room temperature.

#### 2.5.6. Thermostability Analysis

The thermostability of the nanofibrous membranes was investigated by the thermogravimetric analysis (TGA) model (Q5000, TA Instruments, New Castle, DE, USA). The samples were run from 50 to 600 °C, with a heating rate of 20 °C/min under a nitrogen atmosphere. The mass loss of the nanocomposites was recorded against the temperature.

#### 2.5.7. Cytotoxicity Assay

The cytotoxicity test of the nanofibrous membranes was based on the viability of cultured cells on the membranes. Briefly, the growth cells at a density of 5000 cells per well were cultured in a DMEM medium and then cultivated on these square samples (5 mm × 5 mm). They were seeded in the wells of a 96-well tissue culture plate and allowed to attach and grow in wells overnight before being treated. After incubation for another 24 h, the culture medium was removed and replaced with the extraction medium and incubated for 24 h. The viability of cells was analyzed using a Cell Counting Kit-8 (CCK-8) kit, with about 10 µL highly water-soluble tetrazolium-8 (Cell Counting Kit-8) solution in each well (100 µL medium). Meanwhile, it should be incubated for 4 h at 37 °C according to the manufacturer’s instructions (*n* = 5). The cell proliferation without membrane was set as the control. The absorbance was measured at 450 nm for each well by a microplate spectrophotometer.

#### 2.5.8. Antibacterial Assay

The antibacterial activity of each group of samples was detected by the inhibition loop method. The antibacterial activity of the nanofibrous membranes against *Escherichia coli* (ATCC 25922, BLUEFBIO, Shanghai, China) and *Staphylococcus aureus* (CMCC 25923, BLUEFBIO, Shanghai, China) was determined by the agar disk diffusion method and the inhibition zone (mm) was determined. The *E. coli* strain was activated by a diffusion plate method using sterile nutrient agar as a plate, and 1 mL of the bacterial suspension was inoculated, and each contained about 108 cfu/mL. The membranes were cut into about 6 mm^2^ round blocks and placed on inoculated plates, and incubated for 24 h at 37 °C. All samples were sterilized under UV for 3 h prior to adhesion, aiming to directly observe bacterial growth on the plate after incubating the plate at 37 °C for a suitable period of time. The inhibition zone was determined by calculating the diameter of the transparent region that was formed around each nanofiber. The diameter of the inhibition zone was measured by a vernier caliper to obtain the excellent degree of antibacterial property of the membrane with different antibiotic concentrations.

## 3. Results and Discussion

### 3.1. Pre-Characterization

The conductivity of the precursor is displayed in Table S1. As the levofloxacin content increased from 0 wt% to 5 wt%, the conductivity of the gelatin spinning solution was not significantly affected. The surface tension of the gelatin spinning solution was also measured, with a value of 36–38 mN/m. The small molecule levofloxacin had no significant effects on surface tension. In conclusion, the addition of levofloxacin had no obvious effects on the conductivity and surface tension of the precursor.

### 3.2. Morphology of Gelatin/Genipin/Levofloxacin Nanofibers

**Figure 1:**
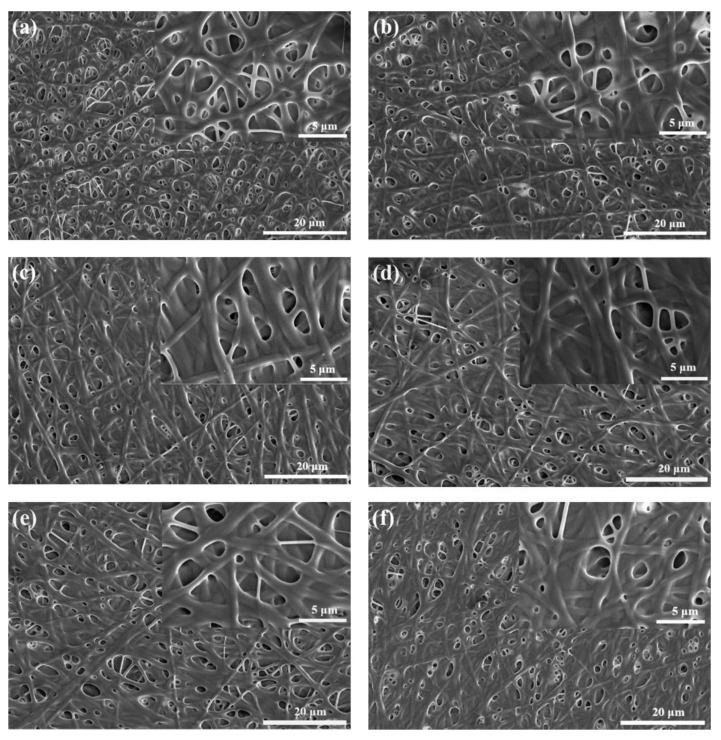
Scanning electron micrograph (SEM) of crosslinked gelatin/genipin fiber membranes with different levofloxacin (Lev) content: (**a**) 0 wt%, (**b**) 1 wt%, (**c**) 2 wt%, (**d**) 3 wt%, (**e**) 4 wt%, (**f**) 5 wt%.

Gelatin/genipin nanofiber membranes with levofloxacin contents of 0 wt%, 1 wt%, 2 wt%, 3 wt%, 4 wt%, and 5 wt% were prepared by electrospinning. Figure S2 provides the photograph of the membrane before crosslinking. For microstructure, Figure 1 shows their SEM images after crosslinking. It was observed that the cross-linked gelatin/genipin nanofibers without levofloxacin had uniform morphology but had no bead shape and dense fiber density (Figure 1a). Furthermore, the membrane shows partly fusion, which is mainly caused by the high-humidity environment during the crosslinking. After the antibiotic levofloxacin was added to the gelatin/genipin system, no obvious granules were observed on the electron micrograph (Figure 1b–f), which indicates that the levofloxacin dissolved well in the gelatin spinning solution system before spinning. Furthermore, levofloxacin does not affect the fine and dense three-dimensional fiber structure of the gelatin/genipin membrane. Moreover, the diameter data of gelatin/genipin fibers with different levofloxacin content is still distributed in the range of 700–900 nm, which suggests that the nanofibrous membrane of gelatin/genipin/levofloxacin has been successfully prepared. All of these could provide a prerequisite for subsequent cell experiments and antibacterial performance tests.

### 3.3. Fourier Transform Infrared Spectroscopy

**Figure 2:**
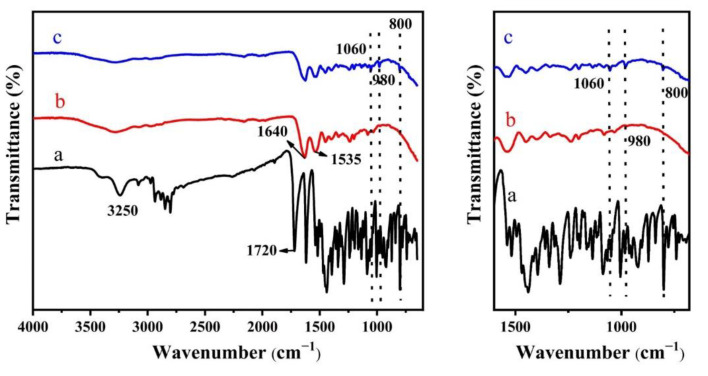
Infrared spectra of levofloxacin and different levofloxacin-containing gelatin fiber membranes: (**a**) levofloxacin drug, (**b**) crosslinked gelatin/genipin membrane, (**c**) crosslinked gelatin/genipin membrane with 5 wt% levofloxacin.

The nanofibrous membranes have been characterized by FT-IR to confirm their chemical structure. Figure 2a is an infrared spectrum of levofloxacin. The peak of 3100–3500 cm^−1^ is caused by the superposition of N-H and O-H stretching vibrations. The 1720 cm^−1^ is the stretching vibration characteristic peak of C=O. Figure 2b shows the spectrum of a crosslinked gelatin/genipin membrane. The wide peak at 3000–3400 cm^−1^ is N-H and O-H vibration. It was also obvious that a peak appeared at 1640 cm^−1^, which is the amide I band of C-N, and the peak at 1535 cm^−1^ is the amide II band from the N-H stretching and C-N bending vibration of genipin. Figure 2c is the spectrum of crosslinked gelatin/genipin membrane with 5 wt% of levofloxacin being added. It can be observed that a weak peak was observed at 1060 cm^−1^, which is caused by the F-C stretching vibration of levofloxacin; in addition, the bands of 980 cm^−1^ and 800 cm^−1^ (benzene ring) were also present [33].

### 3.4. Water Contact Angles

**Figure 3:**
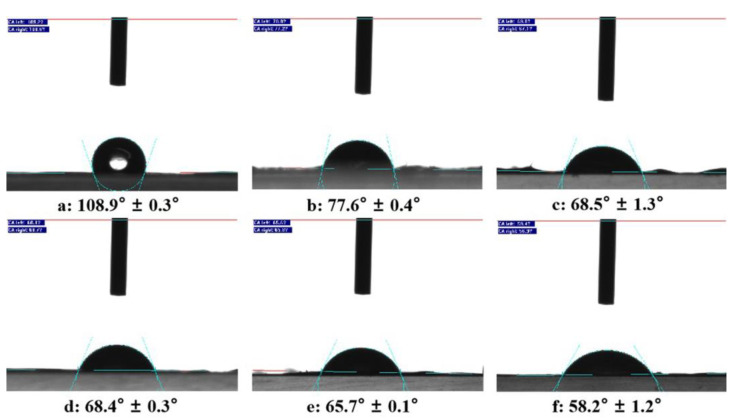
Static water contact angle of gelatin/genipin membranes with levofloxacin content in (**a**) 0 wt%, (**b**) 1 wt%, (**c**) 2 wt%, (**d**) 3 wt%, (**e**) 4 wt%, (**f**) 5 wt%.

As for tissue engineering scaffolds, hydrophilicity is an important factor that affects the cytocompatibility of biomaterials. Figure 3 exhibits the static water contact angle of gelatin/genipin membranes with different levofloxacin content. The contact angle of the cross-linked gelatin/genipin membrane without levofloxacin is about 108.9 ± 0.3°, which shows its hydrophobicity. However, after adding levofloxacin, the stepwise decreased contact angle in Figure 3b–f indicates that the addition of levofloxacin improves the hydrophilic properties of membranes. Many studies have demonstrated that cells adhere, spread, and grow more easily on moderately hydrophilic scaffolds than that on hydrophobic or very hydrophilic ones [18]. Therefore, the addition of levofloxacin is beneficial for improving the hydrophilic property of gelatin/genipin membranes.

### 3.5. Mechanical Properties

**Figure 4:**
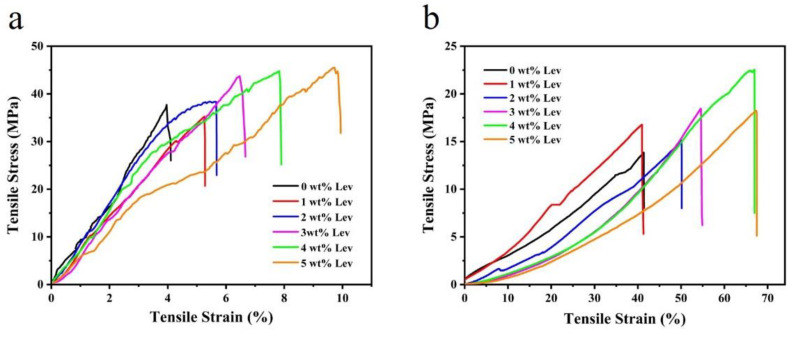
Typical stress-strain curve of gelatin/genipin/levofloxacin membranes in dry state (**a**) and wet state (**b**).

In this experiment, the mechanical tensile test was performed on dry and wet gelatin/genipin/levofloxacin nanofiber membranes because the TM repair underwent a wet transition during use. Figure 4a shows the typical stress-strain curves of the membranes (dry) with a different levofloxacin content. With the addition of levofloxacin, tensile strength and elongation at the break of the membranes have increased. Similar results are also exhibited in the wet membranes and are more pronounced, as shown in Figure 4b. Table 1 displays the date of tensile strength and elongation at the break of the membranes in dry/wet state. At the same time, it also exhibits enhanced tensile strength and elongation at the break when levofloxacin content increases. The introduction of the polar group -F may increase the intermolecular force and strength, as seen in the structure of levofloxacin (Figure S3, which has been added in the Supplementary Materials) [34]. Therefore, the addition of levofloxacin increased the intensity slightly.

By comparing the mechanical data of dry and wet membranes, it can be clearly seen that the wet membranes have a significant decrease in tensile strength, which is about 35–50% of the dry membranes, while the elongation at break increases 7–10 times than the dry membranes. This is because the binding energy of cross-linked gelatin/genipin film can be effectively reduced by micro-immersion in PBS under the action of small molecule polar plasticizer water. On the other hand, when water molecules are absorbed by the membrane, gelatin chains tend to self-extend due to the affinity of water molecules. The water molecules entering the cross-linked network of the membrane cause the swelling of the cross-linked membrane, which will lead to a decrease in mechanical strength and an increase in elongation at the break of the membrane [35]. This can also be confirmed by the change in the fiber morphology of the membrane before and after crosslinking. Moreover, the wet membranes have a tensile strength of 13 to 22 MPa, which is higher than the human TM specimen of 0.6 to 1.0 MPa [20]. Consequently, in mechanics performance, the gelatin/genipin/levofloxacin nanofibrous membranes are suitable for TM patches.

### 3.6. Cell Viability and Proliferation

**Figure 5:**
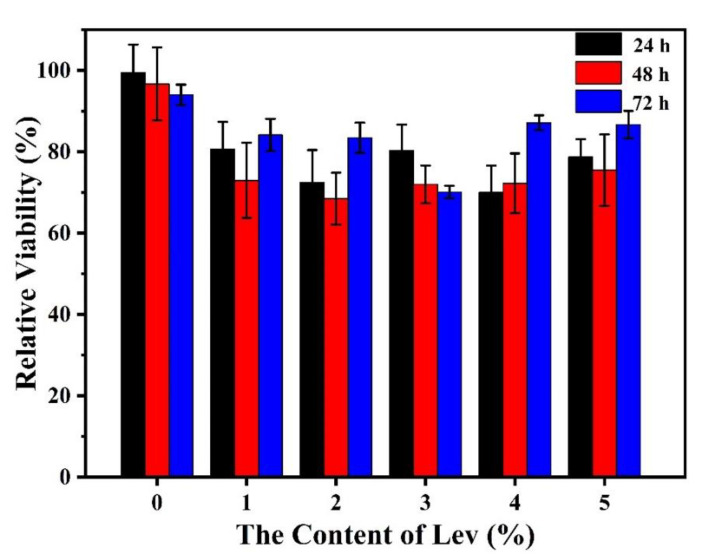
Analysis of relative growth activity of fibroblasts on the gelatin/genipin membranes with different levofloxacin content.

Cell viability experiments were performed on the membranes using fibroblasts that are component parts of a human TM. According to Figure 5, the cells have adhered and proliferated on the gelatin/genipin membranes. This result is similar to our previous work [14], which means the scaffolds can provide an environment to support the formation of mineralized tissue. However, with the addition of levofloxacin, the membranes show an inhibitory effect on the cells at 24 h. It was found that the relative growth activity of fibroblasts decreases based on the five groups of gelatin/genipin/levofloxacin, which indicates that the addition of levofloxacin is not beneficial to the growth of fibroblasts at 24 h. Figure S4 provides the SEM photographs of human skin fibroblasts fixation inoculated on crosslinked gelatin/genipin membrane with 5 wt% levofloxacin for two days. In comparison, after 72 h of cultivation, the relative growth activity of most groups has increased. Therefore, the gelatin/genipin/levofloxacin still has the function of supporting cellular activity over a long culture time.

### 3.7. Antibacterial Properties

**Figure 6:**
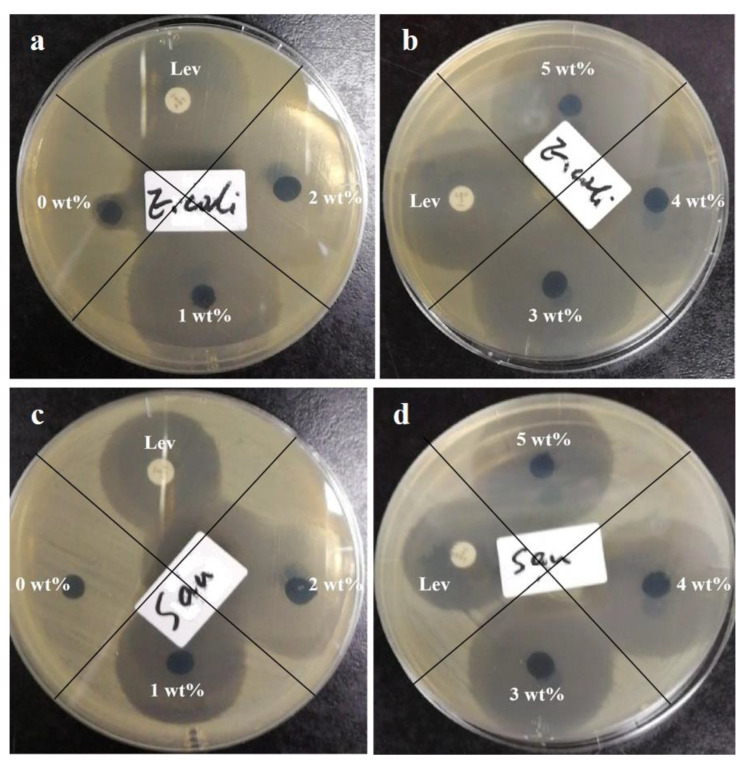
Antibacterial circle diagram of gelatin/genipin/levofloxacin membranes in (**a**,**b**) *Escherichia coli* (*E. coli*) and (**c**,**d**) *S. aureus* (*Sau*).

Antibacterial activity of gelatin/genipin/levofloxacin membranes was detected by conducting the inhibition zone method. Figure 6 exhibits the visual images of the samples against *Escherichia coli* and *S. aureus*. It is obvious that the gelatin/genipin membrane without the antibiotic levofloxacin only maintains the original diameter of the cut (Table 2). When the levofloxacin content was added to 1 wt%, the diameter of the inhibition zone increased sharply to 34 mm and 30 mm, which was comparable to the control group of 5 µg levofloxacin. With the further increase in the amount of levofloxacin, the effects of inhibition were not enhanced obviously. In other words, when the levofloxacin content was only 1 wt%, the prepared gelatin/genipin membranes showed excellent antibacterial properties.

## 4. Conclusions

The preparation of an antibacterial scaffold for TM repair presented in this study used electrospinning gelatin nanofiber membranes that are crosslinked by genipin and contained a different content of levofloxacin were prepared and characterized. SEM proved that the addition of levofloxacin did not affect the nanofiber structure of the cross-linked gelatin/genipin membrane. Furthermore, it was found that the addition of levofloxacin can not only enhance the mechanical properties of the gelatin/genipin membrane but also increase its hydrophilic properties. This improvement to hydrophilicity is more conducive to promoting cell adhesion. On the other side, the results of the antibacterial test revealed that the prepared membrane has excellent antibacterial properties against Gram-negative bacteria *Escherichia coli* and Gram-positive bacteria *S. aureus* when the levofloxacin content is only 1 wt%. The viability and proliferation of fibroblasts on gelatin/genipin/levofloxacin membranes indicated that the scaffolds could support cellular activity in a long culture time. Therefore, compared with different amounts of the additional levofloxacin, the gelatin/genipin membrane with 1% levofloxacin is more suitable for TM repair.

## Figures and Tables

**Table 1:** Tensile strength and elongation at break data for gelatin/genipin/levofloxacin membranes (dry/wet state).

Levofloxacin Content (wt%)	0	1	2	3	4	5
(Dry state) Tensile strength (MPa)	36.8 ± 3.32	35.2 ± 3.14	38.3 ± 2.71	43.4 ± 4.04	44.8 ± 1.34	45.4 ± 3.54
(Wet state) Tensile strength (MPa)	13.8 ± 0.87	16.7 ± 1.43	15.1 ± 1.16	18.4 ± 2.58	22.5 ± 3.74	18.2 ± 2.12
(Dry state) Elongation at break (%)	4.01 ± 0.12	5.25 ± 0.17	5.67 ± 0.14	6.47 ± 0.53	7.32 ± 0.47	9.82 ± 1.01
(Wet state) Elongation at break (%)	41.4 ± 4.12	41.1 ± 3.56	50.1 ± 5.03	54.5 ± 4.18	66.9 ± 6.47	67.3 ± 5.28

**Table 2:** Table of inhibition zone diameters of gelatin/genipin/levofloxacin membranes against *Escherichia coli* and *S. aureus*.

Levofloxacin Content	5 μg	0 wt%	1 wt%	2 wt%	3 wt%	4 wt%	5 wt%
*Escherichia coli*	34 mm	8 mm	34 mm	36 mm	35 mm	35 mm	36 mm
*S. aureus*	29 mm	6 mm	30 mm	31 mm	33 mm	33 mm	33 mm

## Data Availability

The data are included in this manuscript and in its Supplementary Materials.

## Supplementary Materials

The following supporting information can be downloaded at: https://www.mdpi.com/article/10.3390/molecules27092906/s1, Figure S1: The photograph of the membrane before (left) and after crosslinking (right); Figure S2: Scanning electron micrograph and distribution of fiber diameter of gelatin fiber membranes with different levofloxacin (Lev) content before the crosslinking: (a) 0 wt%, (b) 1 wt%, (c) 2 wt%, (d) 3 wt%, (e) 4 wt%, (f) 5 wt%; Figure S3: Chemical structure of levofloxacin; Figure S4: SEM photographs of human skin fibroblasts fixation inoculated on crosslinked gelatin/genipin membrane with 5 wt% levofloxacin for 2 days; Table S1: Conductivity and surface tension data of different levofloxacin content gelatin spinning precursor.

## Sample Availability

Samples of the compound are available from the authors.
