# Supplementary material for: Preparation of Antibacterial Gelatin/Genipin Nanofibrous Membrane for Tympanic Membrane Repair
Source: Molecules. 2022 May 3;27(9):2906. doi: 10.3390/molecules27092906 (PMC9104484; doi:10.3390/molecules27092906)
Supplement: Supplementary file 1 [file molecules-27-02906-s001.zip › molecules-1662978-supplementary.pdf]

molecules

Supplementary Material

Preparation of Antibacterial Gelatin/Genipin Nanofibrous Membrane for Tympanic Membrane Repair

Shuying Han [1], Zhaohua Zhang [1], Jia Chen [2], Jie Li [3], Mi Zhou [1,*], Zejian He [1], Zhen He [1] and Longfei Li [1]

1 Material Science and Engineering, College of Materials Science and Engineering, Zhejiang University of Technology, Hangzhou 310000, China; 17826801650@163.com (S.H.); zhaohua119@126.com (Z.Z.); hezejian@zjut.edu.cn (Z.H.); hezhen0821@163.com (Z.H.); lilongfei9708@163.com (L.L.)
2 The Department of Otolaryngology, The Second Affiliated Hospital, School of Medicine, Zhejiang University, Hangzhou 310000, China; chenjia212c@zju.edu.cn
3 Research and Development Department, Hangzhou Singclean Medical Products Co., Ltd., Hangzhou 310000, China; lijie@hzxhe.com
* Correspondence: zhoumi@zjut.edu.cn

Instrumentation

Morphological Characterization

The morphology of and gelatin nanofibers was investigated by scanning electron microscopy (SEM, S-4700, Hitachi, Japan). The randomly selected fiber areas were cut into squares and coated with a thin layer of gold. The diameter of nanofibers was determined by using the image analysis software, Nano Measurer. 50 different nanofibers were randomly measured from SEM images and the average diameter of the nanofiber membranes was analyzed.

Culture and Adhesion

For the investigation of human skin fibroblast cells' morphology and their adhesion, the crosslinked membranes were cut into a square of 1 cm×1 cm and sterilised with 75% alcohol for 1 h. The growth cells in DMEM medium were then cultivated on these square samples and cell detachment was investigated. Human skin fibroblast cells with 3×10^5 cells/well was seeded on the prepared square samples. After culture for 48h, the samples were fixed with glutaraldehyde 2.5% overnight at 4 ℃. From then onwards the samples were dehydrated ethanol/distilled water from 30 to 100% in steps of 10% at 15 min intervals. The dried samples were coated with gold by sputtering for further cell morphology analysis on the surface of the coatings by FE-SEM (SU-70, Hitachi, Japan) at 2500× maganification.

Conductivity and Surface Tension

The surface tension of the precursor solution was checked by pendant drop method on a video-based optical contact angle meter (Dataphysics OCA-20, Germany). The solution drop volume of the test was controlled by a micro syringe and the test condition was at room temperature. At least 3 times were measured and averaged on each sample surface. The conductivity of the solution was tested using a digital con-ductivity meter (Lei magnetosound Instrument, DDS-11A, Shanghai).

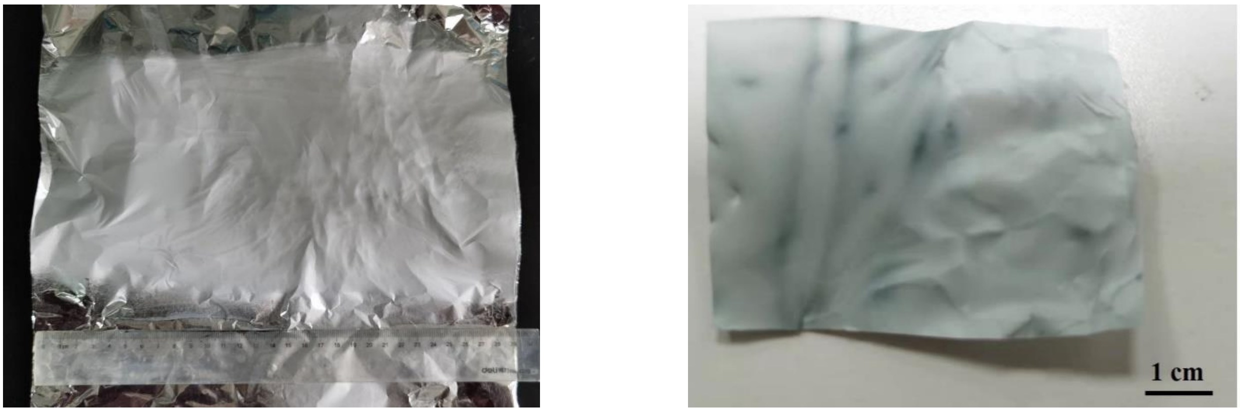

Figure S1. The photograph of the membrane before (left) and after crosslinking (right).

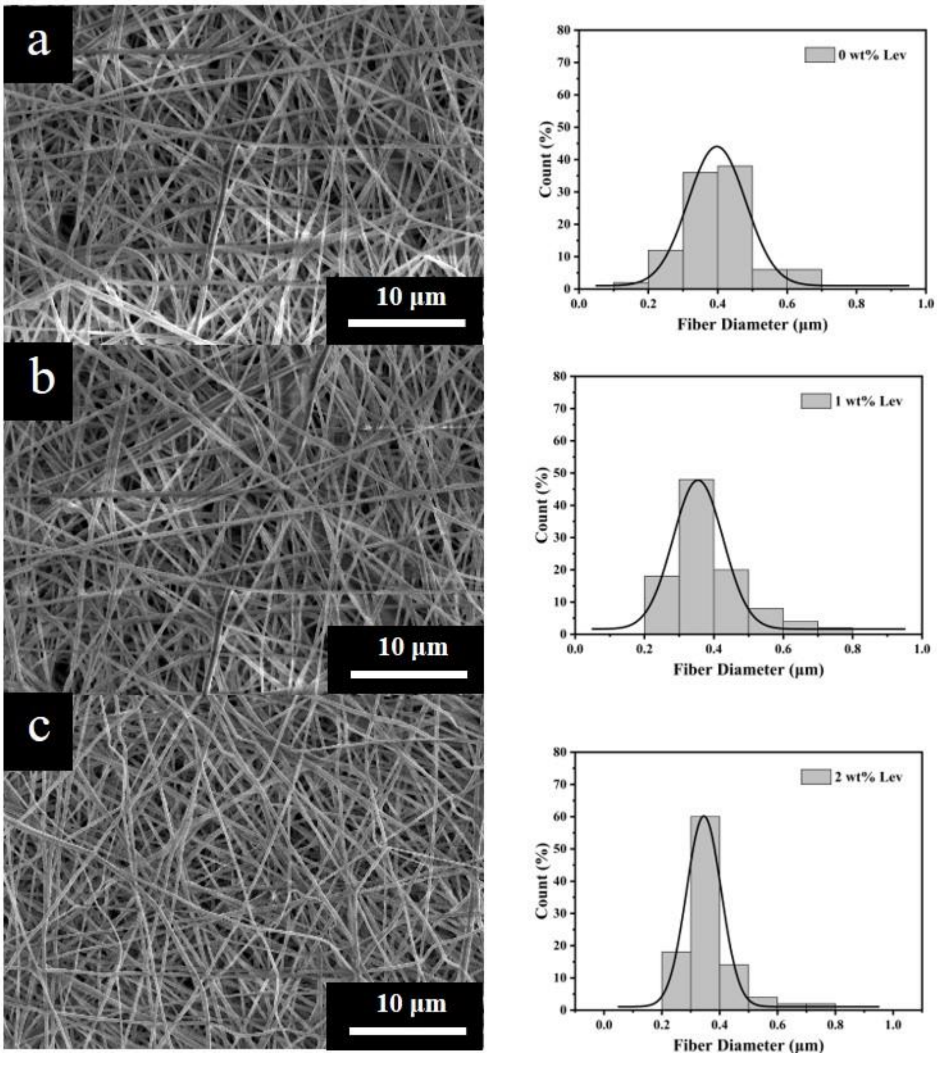

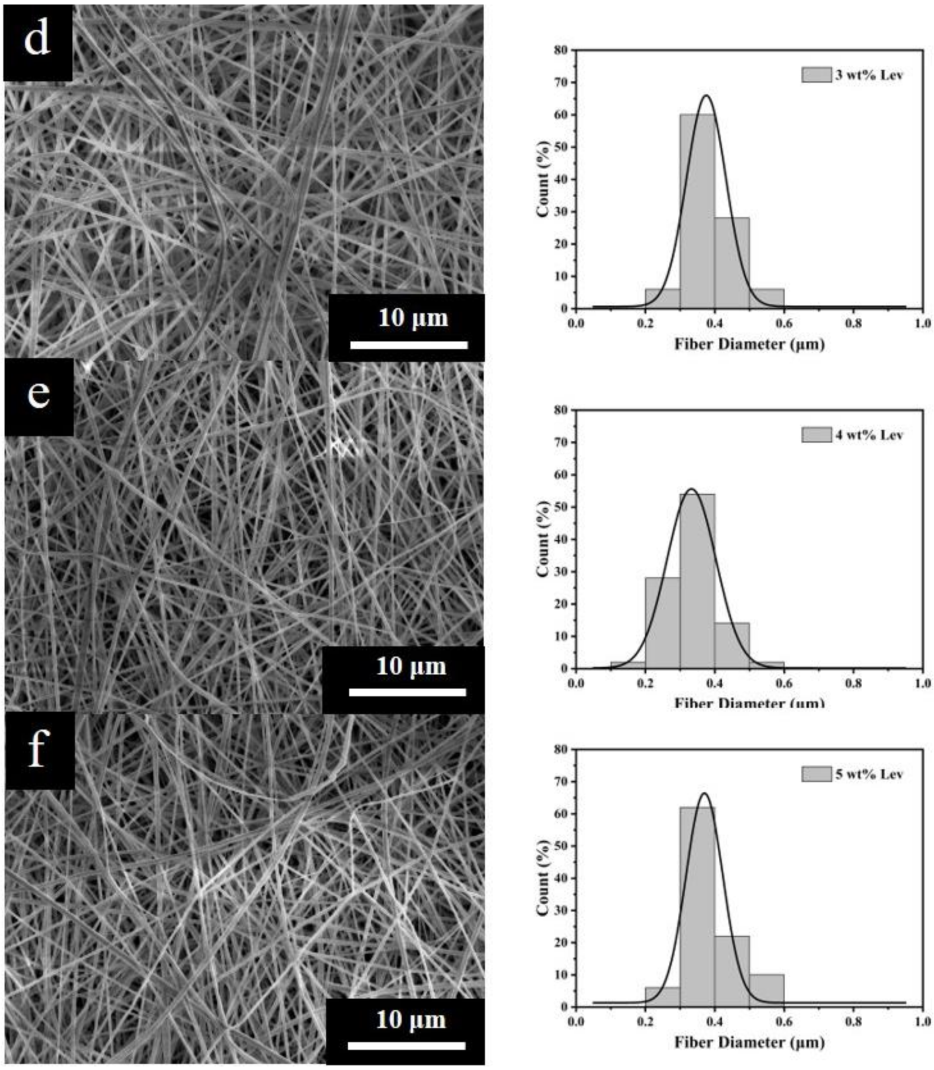

Figure S2. Scanning electron micrograph and distribution of fiber diameter of gelatin fiber membranes with different levofloxacin (Lev) content before the crosslinking: (a) 0 wt%, (b) 1 wt%, (c) 2 wt%, (d) 3 wt%, (e) 4 wt%, (f) 5 wt%.

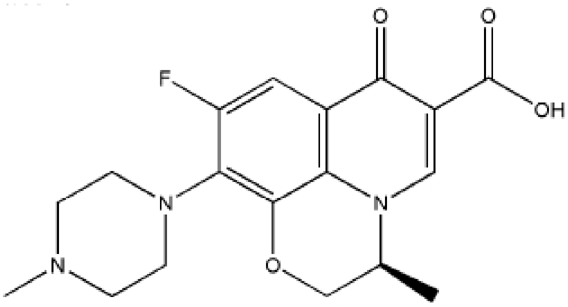

Figure S3. Chemical structure of levofloxacin.

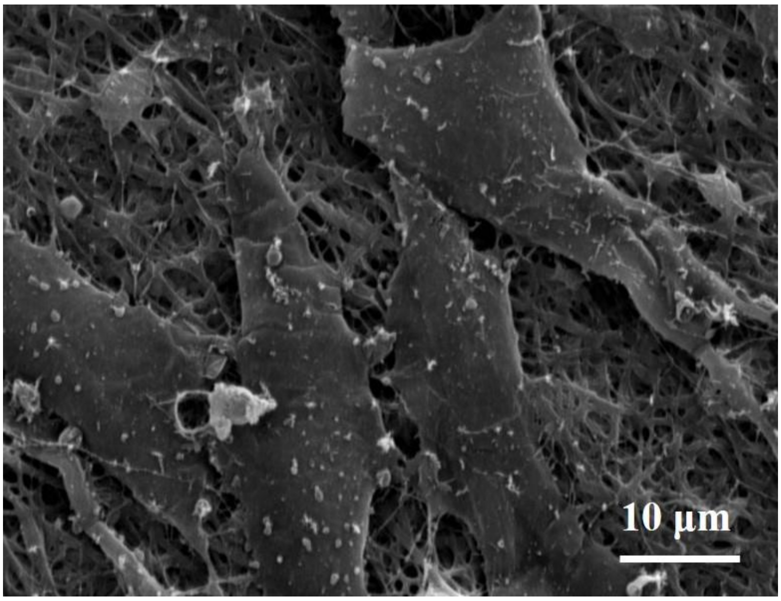

Figure S4. SEM photographs of human skin fibroblasts fixation inoculated on crosslinked gelatin/genipin membrane with 5 wt% levofloxacin for 2 days.

Table S1. Conductivity and surface tension data of different levofloxacin content gelatin spinning precursor.

**Levofloxacin content (wt%)**	**0**	**1**	**2**	**3**	**4**	**5**
Conductivity (ms/cm)	2.18 ± 0.05	2.35 ± 0.03	2.39 ± 0.04	2.41 ± 0.03	2.30 ± 0.02	2.33 ± 0.01
Surface Tension (mN/m)	36.26 ± 0.32	37.18 ± 0.32	36.59 ± 0.22	37.83 ± 0.49	36.73 ± 0.34	36.54 ± 0.51
